# Is experiential-intuitive cognitive style more inclined to err on conjunction fallacy than analytical-rational cognitive style?

**DOI:** 10.3389/fpsyg.2015.00085

**Published:** 2015-02-06

**Authors:** Yong Lu

**Affiliations:** Construction Management Department, University of Shanghai for Science and TechnologyShanghai, China

**Keywords:** conjunction fallacy, cognitive style, individual differences, information processing

## Abstract

In terms of prediction by Epstein’s integrative theory of personality, cognitive-experiential self-theory (CEST), those people with experiential-intuitive cognitive style are more inclined to induce errors than the other people with analytical-rational cognitive style in the conjunction fallacy (two events that can occur together are seen as more likely than at least one of the two events). We tested this prediction in a revised Linda problem. The results revealed that rational and experiential cognitive styles do not statistically influence the propensity for committing the conjunction fallacy, which is contrary to the CEST’s predictions. Based on the assumption that the rational vs. experiential processing is a personality trait with comparatively stabile specialities, these findings preliminarily indicate that those people who are characterized by “rational thinking” are not more inclined to use Bayes’ deduction than the other people who are labeled by “intuitive thinking” or by “poor thinking.”

## INTRODUCTION

Traditional assumptions about rationality presume that when people deduce, their judgment should abide by Bayes’ Rule (Morris, 1974, 1977). However, since Simon (1957) proposed the idea of *bounded rationality*, a significant amount of experimental and field evidence suggests that people sometimes do not perform perfectly due to inner or outer factor restrictions such as cognitive limitations, logical errors, misapprehensive implication, pressured time allocation, or varying contents. In particular, studies based on probability judgment suggest that people often show biases (e.g., base-rate neglect, conjunction fallacy, disjunction fallacy, hindsight bias, overconfidence, and sample-size neglect) in the probabilistic (Bayesian) inference tasks and therefore violate some fundamental key properties of classical probability theory. Among these biases, the conjunction fallacy is a related phenomenon that has been well-researched in the past 30 years and becomes one necessary component in bias and fallacy studies.

### CONJUNCTION FALLACY AND COGNITIVE STYLES

The *conjunction fallacy* explores how individuals commonly violate a basic probability rule by estimating probability of conjunction of two statements to be more probable than the probability they assign to at least one of its constituent statements. Tversky and Kahneman (1983)first proposed the conjunction fallacy. In their seminal study, they presented participants with an afterward well-known probability judgment scenario named the LINDA task. A hypothetical woman named Linda as well as a personality sketch on some of her characteristic and activities functioned as the target *E* on which they later asked the participants to make judgment about Linda.

(*E*) *Linda is 31 years old, single, outspoken, and very bright. She majored in philosophy. As a student, she was deeply concerned with issues of discrimination and social justice, and also participated in antinuclear demonstrations.*

After reading the description of that target *E*, they requested the participants to estimate the probability of a number of statements that were true referring to *E*. Three statements are included as follows:

(*T*) *Linda is a bank teller.*
(*F*) *Linda is active in the feminist movement.*
(*T ∧ F*) *Linda is a bank teller and is active in the feminist movement.*

The participants should estimate the individual statement *T* as more likely than the conjunction *T* ∧*F* since it is impossible for Linda to be a feminist bank teller without also being a bank teller. However, a significant amount of later studies found that the majority of respondents commit single conjunction fallacy and even double conjunction fallacy (e.g., Abelson et al., 1987; Fantino et al., 1997). The single conjunction fallacy means that respondents judge the conjunctive estimate being higher than one of the constituent and being lower than the other constituent. The double conjunction fallacy means that respondents judge the conjunctive estimate being higher than both of the constituents. The observed high error rate is most extraordinary and was even up to 87% in Tversky and Kahneman (1983). Strikingly, even in a distinct, succinct Linda problem in which only three statements *T*, *F*, and *T* ∧*F* were presented to participants, it was still found in Tversky and Kahneman (1983) that 85% of the participants rated *T* ∧*F* as more probable than *T*. The accumulated evidence of experimentation from 1980s has suggested that the violation is highly robust to variations in response modes and is very easy to replicate in a variety of contexts.

Major theoretical interpretations postulate that the conjunction fallacy is unquestionably considered a probabilistic error (e.g., Tversky and Kahneman, 1983; Bar-Hillel and Neter, 1993; Costello, 2009) or linguistic misapprehension (e.g., Wolford et al., 1990; Politzer and Noveck, 1991; Hertwig et al., 2008; Hartmann and Meijs, 2012). For instance, Costello (2009) proposed that participants represent the conjunction as an effect of random error in the judgment process. Politzer and Noveck (1991) argued that the task demands are likely to compel participants to misinterpret a base statement (e.g., *T*) as the conjunction of the base statement and the complement of an added statement [e.g., *T* ∧¬*F* (Linda is a bank teller and she is not a feminist)]. Therefore, when assuming that *T* and *F* are independent and that participants’ probability estimate on *F* is larger than 0.5, the probability of *T* ∧*F* is larger than the probability of *T* ∧ ¬*F*. However, Moro (2009) provided indirect support for the reasoning bias hypothesis against the misunderstanding hypothesis (also see, Wedell and Moro, 2008). Furthermore, Tentori and Crupi (2012; also see, Tentori et al., 2004) obtained results overtly contradictory to the claims of Hertwig et al. (2008, p. 740) that the conjunction fallacy should be instead considered as reflecting “reasonable pragmatic and semantic inferences.” In short, the misunderstanding explanation attributes the conjunction and disjunction fallacies to people’s mistake on comprehending the connecting words or the statements.

On the other hand, some critical explanations that have been proposed based on Bayesian solutions suggested that the conjunction fallacy might not be fallacious in certain circumstances (among others Bovens and Hartmann, 2003; Busemeyer et al., 2011; Tentori et al., 2013). For instance, Bovens and Hartmann (2003) explicitly argued from source reliability perspective that the conjunction fallacy can be accounted for in a Bayesian framework given prior beliefs in the likelihood of Linda being a feminist given her background description. They argue that participants who believe *T* ∧*F* more than *T* are rational if and only if: Δ*Prob* = *Prob*(*T*, *F*|Rep*T*, Rep*F*) – *Prob*(*T*|Rep*T*) > 0, where Rep*T* denotes a report of *T* by the participants’ certain witness scenario. If so, the respondents would, in a Bayesian perspective, not be committing a reasoning fallacy when responding that *T* ∧*F* is more likely than *T*. Busemeyer et al. (2011) proposed that in accordance with a generalization of Bayesian probability theory, quantum probability model can explain the conjunction fallacy, though Tentori and Crupi (2013) argued against their approach’s explanation. Tentori et al. (2013) put forth new empirical findings as defined by contemporary Bayesian theory of argument that the conjunction fallacy depends on the added conjunct (e.g., *F*) being perceived as *inductively confirmed* rather than some of competing explanations’, e.g., the averaging hypothesis (Fantino et al., 1997), the random error model (Costello, 2009), proposals that the conjunction fallacy rates would rise as the *posterior probability* of the added conjunct does. Tentori et al. (2013) argued that their results cannot be explained by those prevalent judgment models and provide new evidence for the role of inductive confirmation as a major determinant of the conjunction fallacy.

A third perspective postulates that the conjunction fallacy is aroused by incorrectly using certain integrate computing models (compensatory strategies) or heuristic models (non-compensatory strategies) (see Gavanski and Roskos-Ewoldsen, 1991 and Betsch and Fiedler, 1999 for a discussion). These integrate computing models include proposals as diverse as averaging rule hypotheses (Fantino et al., 1997), configural weighted average model (Juslin et al., 2009; Nilsson et al., 2009), conjunction coefficient model (Abelson et al., 1987), fuzzy logical model of perception (Massaro, 1994), random error model (Costello, 2009), and signed sum model (Yates and Carlson, 1986). For instance, Fantino et al. (1997) proposed that people non-normatively average the likelihood of the two components in arriving at a judgment of the likelihood of the conjunction. At the same time, these heuristic models include proposals such as potential surprise hypothesis (Fisk, 2002) and the equate-to-differentiate model (Lu, under review). For instance, Lu (under review) assumes that people implement a judgmental process by filtering one or several less distinct (or “futile”) dimension(s) of each propositional statement (e.g., T of *T* and T of *T* ∧*F*, where T denotes *T*’s one dimension) and base their judgments of the relative likelihoods of the joint and single statements on the values derived from the most distinct dimension. One statement with a larger outcome of its reserved dimension is preferred to another statement with a less outcome of its reserved dimension.

The question of whether people rely on Bayesian rules (no matter whether Bayesian probability theory is believed in the correct way or other kind of understandings), the integrative models, or the heuristic models in their judgment is still in dispute (e.g., Denes-Raj and Epstein, 1994; Kemmelmeier, 2009). However, recent studies, (e.g., Brandstätter et al., 2006; Birnbaum and LaCroix, 2008; Wang and Li, 2012) have accumulated some evidence that supports for the heuristic models rather than the integrative or Bayesian models. As the substitutive rules to compensate people’s incorrect use of Bayes’ Rule, those integrative models’ explanations, e.g., the weighting and summing calculation process (Nilsson et al., 2009), assume as usual as the expectation rule that people should be competent for the needed quantitative calculation. By contrast, the heuristic models’ interpretations shed light on people’s non-compensatory strategies and propose a bounded rationality perspective on the phenomena. Furthermore, the heuristic models highlight more closely people’s fundamental and underlying cognitive processes than the integrative models that emphasis on outcome prediction or goodness-of-fitting. The attempt to employ the heuristic models for modeling cognition has enabled the introduction of several new concepts in psychology, such as simple heuristics, ecological or pragmatic rationality, and bounded rationality. The existing formal Bayesian frameworks (notably classical probability theory) are more and more questioned, and some tendencies have been gained to employ these heuristic conceptual approaches to veritably model human judgment illusions under uncertainty, such as the conjunction fallacy (for the integrative vs. heuristic models’ debate, also see, Gigerenzer and Selten, 2001 for a discussion).

A number of studies found that a range of boundary factors, e.g., individual differences of cognitive ability, cognitive styles, causal reasoning, numeracy measures, and cognitive reflection (among others Epstein et al., 1995; Stanovich and West, 1998; Fisk, 2005; Feeney et al., 2007; Morsanyi et al., 2010), influence the incidence of the conjunction fallacy. For instance, Epstein et al. (1995) demonstrated that the influence of statistical sophistication may slightly account for the conjunction fallacy, though concrete and unnatural context is ascribed to the primary reason. Feeney et al. (2007) measured participants’ cognitive ability concerning judgment of numerical series, verbal analogies, arithmetic computations, superimpositions and so on and found an association with ability for the categorical materials in the conjunction fallacy. Fisk (2005) investigated how working-memory (reading and computation span, information-processing speed, and progressive matrices) and intellectual functioning influence on the conjunction fallacy. However, the results generally found a paradoxical tendency for higher levels of intellectual and working-memory functioning to be associated with lower levels of accuracy on the conjunction fallacy except that in one Bayesian judgment, participants who scored high on the working-memory measures are more accurate. Stanovich and West (1998) confirmed that participants of higher cognitive ability are disproportionately likely to avoid the conjunction fallacy.

The *cognitive style* is one of the individual difference variables that affects one’s performance and more specifically, the cognitive processing in judgmental decisions. Messick (1976) characterized cognitive styles as stable attitudes, preferences, or habitual strategies that determine individuals’ modes of perceiving, remembering, thinking, and problem solving. Kozhevnikov (2007) defined cognitive styles as representing heuristics that individuals use to process information.

Epstein (1990, 1994, 2003; also see, Kirkpatrick and Epstein, 1993; Epstein et al., 1996, 1999; Pacini et al., 1998; Epstein and Pacini, 2001; Norris and Epstein, 2011) posited that according to their cognitive-experiential self-theory (CEST), people process information by two likely parallel but interacting modes of cognitive styles: analytical-rational style and experiential-intuitive style. In general, the analytical-rational style operates primarily at the conscious level and is computative, intentional, analytic, logical, and relatively affect-free. In contrast, the experiential-intuitive style is a relatively crude system and is assumed to be automatic, more rapid processing oriented, and associative. Epstein (2003) assumes that the analytical-rational style has capability to understand and correct for the procedure of the experiential-intuitive style, however, the latter style is unaware and independent of the former style. As a result, those who are prone to endow with experiential-intuitive style are capable of eliminating its influence when they are aware to behavior more rationally. However, no evidence ever supports the existence of two totally unrelated cognitive styles. It is more probable that maybe a single bipolar information processing system of intuition-analysis governed by a common set of principles works on people’s daily cognitions (Hayes et al., 2003).

Similar to the integrate computing models versus the heuristic models, the analytical-rational and experiential-intuitive cognitive styles can be synonymously seen as based on dual-process theories from social psychology (see, Chaiken and Trope, 1999). Although the two cognitive styles, the two models, and other similar theories (such as Chaiken and Trope’s (1999) heuristic-systematic model and Bargh et al.’s (2001) automatic vs. non-automatic processing) are different in their labeling of the two processes (e.g., computing vs. heuristic, rational vs. intuitive, heuristic vs. systematic, automatic vs. non-automatic, etc.), they share common two systems as described by the two cognitive styles.

It is different from other dual-process models in that first, the CEST put a dual-process procedure in personality traits and unify them into one organized adaptive processing, rather than considering it as a separate cognitive constructs in the context of other theories (Pacini and Epstein, 1999). Cognitive styles in preference for rational or intuitive processing are therefore defined and can also be measured by the CEST. Second, the CEST presents an adaptive unconscious system in the experiential-intuitive style according to an evolutionary perspective. Third, the CEST gains an understanding on how the analytical-rational and experiential-intuitive cognitive styles can lead to human irrational behaviors (e.g., Denes-Raj and Epstein, 1994).

According to the CEST’s hypothesis, certain people with the experiential-intuitive thinking style were likely to commit the conjunction fallacy, while compared to the idea that other people with the analytical-rational thinking style were likely to avoid the conjunction fallacy. On the other hand, according to the second explanations’ criticism that there is no conjunction fallacy at all, therefore, there should be no correlation between the experiential-intuitive thinking style and the conjunction fallacy. At the same time, there should be no correlation between the analytical-rational thinking style and no conjunction fallacy.

Although some studies have specifically addressed the correlation between the CEST and the tendency to commit the conjunction fallacy (see, Epstein et al., 1995, 1999), there is limited research if any that directly sheds light on how cognitive styles influence on the conjunction fallacy. Epstein et al.’s (1995) study focused on the influence of context that based on natural-unnatural and concrete-abstract dimensions. In the study by Epstein et al. (1999), the degree to which people believe a representativeness heuristic or a conjunction-rule provide the more compelling solution to the Linda problem was measured. In sum, Epstein et al. (1999) found that people’s normal response to the Linda vignette from an experiential perspective results to the usually high rate of conjunction fallacy, even though a substantial number of people who know and think of the conjunction rule prefer to use the representativeness heuristic.

In the remainder of this paper in the light of the framework of the CEST, we attempt to address the void about if the assumed rational-experiential cognitive styles lead to the conjunction fallacy. Next, we test this hypothesis.

## EXPERIMENT

### METHOD

#### Participants

Two hundred and fifty Chinese bachelor students of first, second, and third-year grades specializing in Chemistry Engineering at the University of Shanghai for Science and Technology volunteered to take part in the study. The ages of the participants were between 17 and 22 years old, and the mean age was 19.8. The female percentage of the participants was 54%.

#### Design

A within-subjects design was used. We investigated in four variations (groups) of the Linda task assessing various affirmative events and various conjunctions (see **Table 1**, for the conjunctive statements and their constituents used in the stimulus through Group 1–4). Therefore, the assessment of rational-experiential cognitive styles in all the three likelihood types of low–low, high–low, high–high marginal probabilities can be useful to impartially test the CEST’s hypothesis.

**Table 1:** Means and median probability estimates in the experiment.

	Probability estimates	
Items^a^	Mean(%)^b^	Median (%)
**Group 1 (*N* = 104^**c**^)**
*Linda is a bank teller.* (*T*)	13.3 (1.2)	10
*Linda is active in the feminist movement.* (*F*)	78.1 (1.8)	80
*Linda takes Yoga classes.* (*Y*)	42.0 (2.2)	50
*Linda is a teacher in elementary school.* (*P*)	17.7 (1.5)	15
*Linda is a bank teller and is active in the feminist movement.* (*T* ∧*F*)	**30.2 (2.5)**	30
*Linda takes Yoga classes and is a teacher in elementary school.* (*Y* ∧*P*)	**22.3 (2.1)**	15.5
**Group 2 (*N* = 37)**
*Linda is a bank teller.* (*T*)	32.2 (4.2)	30
*Linda is active in the feminist movement.* (*F*)	67.5 (3.8)	70
*Linda is an executive.* (*D*)	39.9 (4.3)	40
*Linda subscribes to a popular liberal magazine.* (*M*)	72.0 (3.8)	80
*Linda is a bank teller and is active in the feminist movement.* (*T* ∧*F*)	**38.6 (4.2)**	40
*Linda is an executive and subscribes to a popular liberal magazine.* (*D* ∧*M*)	**49.8 (4.1)**	50
**Group 3 (*N* = 41)**
*Linda is an avid reader.* (*R*)	72.1 (2,8)	80
*Linda is active in the feminist movement.* (*F*)	72.2 (2.9)	80
*Linda is an executive.* (*D*)	40.0 (3.0)	40
*Linda subscribes to a popular liberal magazine.* (*M*)	68.6 (3.5)	75
*Linda is an avid reader and is active in the feminist movement.* (*R* ∧*F*)	**66.9 (3.1)**	70
*Linda is an executive and subscribes to a popular liberal magazine.* (*D* ∧*M*)	**44.5 (3.3)**	50
**Group 4 (*N* = 42)**
*Linda is a bank teller.* (*T*)	24.3 (3.2)	20
*Linda is very shy.* (*S*)	11.7 (2.3)	7
*Linda is a teacher in elementary school.* (*P*)	43.7 (4.5)	50
*Linda is active in crafts like needlepoint.* (*C*)	31.2 (3.7)	20
*Linda is a bank teller and is very shy.* (*T ∧ S*)	**15.2 (2.8)**	10
*Linda is a teacher in elementary school and is active in crafts like needlepoint.* (*P ∧ C*)	**31.4 (3.6)**	30

^a^In the version given to participants, the labels *P*, *F*, *T*, *Y*, *R*, *S*, *M*, *C*, *D*, *T* ∧ *F*, *Y* ∧*P*, *D* ∧*M*, *R* ∧*F*, *T* ∧*S*, and *P* ∧*C* were omitted.

^b^Standard errors with 95% confidence intervals are in parentheses. Boldface indicates a significant difference, relative to the conjunctions and their corresponding unlikely constituents (*p* < 0.05).

^c^There are so many more participants in Group 1 because the Experiment was conducted firstly through Group 1, however, the likelihood types of the Group 1’s statements are mostly the likelihood type of “Unlikely ∧ Likely” and have not enough data in relation to the types of “Likely ∧ Likely” and “Unlikely ∧ Unlikely.” On the other hand, some studies indicate that the conjunction fallacies are related to the likelihood types (e.g., Tversky and Kahneman, 1983; Yates and Carlson, 1986; Fantino et al., 1997; Nilsson et al., 2009) and that more conjunction fallacies should be happened in the likelihood type of “Unlikely ∧ Likely” rather than the likelihood types of “Unlikely ∧ Unlikely” and “Likely ∧ Likely” (e.g., Yates and Carlson, 1986; Fisk, 1996). Thereof, the influence of the two kind of cognitive styles on the conjunction fallacy may be potentially affected if single likelihood type, such as *T* ∧*L* (mostly this combination is the likelihood type of “Likely ∧ Likely”), rather than all of the three likelihood types is concerned. In order to avoid unbalanced potential variable of the likelihood types’ influence on the Experimental assumption, “Likely ∧ Likely” and “Unlikely ∧ Unlikely” combinations of likelihood types of the statements through latter three Groups are thereafter included. Needed numbers of the latter three Groups’ participants are employed to generate much more needed likelihood types.

#### Materials

The questionnaires contained two pages and consisted of the following two parts:

The first part located at the first page. and presented a modified Linda problem derived from Tversky and Kahneman (1983). For Group 1 to 4, after reading the same personality description of Linda (target *E*), participants in each group were instructed to estimate on probabilities of two conjunctive statements and their respective constituents (see **Table 1**, for the respective stimulus used in each group). The order of the statements was counterbalanced. For each group, the independent variable was the two conjunctive statements and their constituents. Probability judgment and measures derived from these, such as conjunction fallacy, were dependent variables. The participants were invited to use a number in the whole range 0–1 (including 0 and 1) either by decimal, fractional, or percent estimates as a reply to each statement. It was mentioned in the instructions that when they forecast that a statement is *unlikely* to happen, they should determine its probability within the range of 0–0.50, and when they forecast that a statement is *likely* to happen, they should determine its probability within the range of 0.5–1, where 0 means minimal probability and 1 means maximal probability.

The second part located at the second page and presented the Rational-Experiential Inventory-10 items short version (the REI-10; Epstein et al., 1996) with a separate scale for rational and experiential thinking styles, corresponding to analytic and heuristic processing respectively. The REI-10 consists of two sub-scales: need for cognition (NFC; e.g., “I would prefer complex to simple problems”) and faith in intuition (FI; e.g., “My initial impressions of people are almost always right”). The NFC scale is intended to measure an individual’s tendency to enjoy and seek challenging intellectual experiences, while the FI scale is intended to assess an individual’s reliance on experiential processing. Each scale is divided into five statements respectively for NFC and FI. The independent variable is the REI-10. Scores and measures derived from these items are dependent variables. One bilingual person and the experimenter (myself) independently translated all the items into Chinese, and some 80% of the translations were exactly the same; for the rest of 20% both translations were sent to a third person to decide upon the best wording. A check was carried out by translating the Chinese items back into English. The participants through Group 1–4 scored each item on a 5-point scale. Because the rating scales ranged from 1 (*completely false*) to 5 (*completely true*), the higher the numerical rating was, the higher the level of agreement with a scale item. Two of the NFC items were reverse scored.

#### Procedure

The experiment was conducted in four quiet classrooms. As the study presented no more than minimal risk of harm to the participants and involved no procedures for which written consent is normally required outside of the study context, the Ethics Committee specifically approved verbal informed consent being provided to the participants in lieu of signed informed consent. The experimenter (myself) read out loud the verbal informed consent at the outset of the experiment. All of the participants consented to participate in the experiment. After that, the experimenter (myself) read out loud a general instruction to inform the participants that the questionnaires were designed to look for the way that people make their judgments under uncertain situation. Then the experimenter gave each participant one leaflet presented with instructions and questions. Questions were answered individually at the participants’ seats.

### RESULTS AND DISCUSSION

#### Probabilities

Participants whose answers for the first part were incomplete (by omitting probability judgments of at least one statement except for the unrelated statement in each group) were excluded from the analysis (*N* = 26). For the remaining 224 participants, the means and median probability estimates from Group 1–4 for the corresponding two conjunctive statements and their respective constituents are shown in **Table 1**, with SEs (95% confidence interval) in parentheses. Results of paired *t* test showed that the mean estimates for all of the conjunctions through Group 1–4 were significantly greater than the mean estimates for their corresponding unlikely constituents [*t*(103) = -7.723, *p* < 0.05 for *T* ∧*F* and its corresponding unlikely constituent in Group 1; *t*(103) = -4.094, *p* < 0.05 for *Y* ∧*P* and its corresponding unlikely constituent in Group 1; *t*(36) = -1.881, *p* < 0.05 for *T* ∧*F* and its corresponding unlikely constituent in Group 2; *t*(36) = -3.724, *p* < 0.05 for *D* ∧*M* and its corresponding unlikely constituent in Group 2; *t*(40) = -1.275, *p* < 0.05 for *R* ∧*F* and its corresponding unlikely constituent in Group 3; *t*(40) = -1.925, *p* < 0.05 for *D* ∧*M* and its corresponding unlikely constituent in Group 3; *t*(41) = -2.566, *p* < 0.05 for *T* ∧*S* and its corresponding unlikely constituent in Group 4; *t*(41) = -3.066, *p* < 0.05 for *P* ∧*C* and its corresponding unlikely constituent in Group 4], as shown in bold on significant effects.

#### Components and conjunction fallacy

A weighted average of 49.78% single conjunction fallacy (min 34.2%, max 64.5%) and a weighted average of 16.7% double conjunction fallacy (min 7.1%, max 31.7%) were made. Totally the participants made a weighted average of 66.48% single and double conjunction fallacies.

#### Individual differences

For the NFC sub-scale, there were 219 participants included in the analysis and 217 included for the FI sub-scale. As five participants did not give rating for any of the items in the REI-10 and two participants only responded to the NFC sub-scale and four items of the FI sub-scale, the data for the left item could not be identified in the pre-testing data set. The mean score for the Rational scale was 2.7 (SD = 0.65) and for the Experiential scale was 3.2 (SD = 0.67). Analyses of the inter-correlations between the REI-10 sub-scales essentially replicated those of Epstein et al. (1996) in that the NFC and FI scales showed no correlation (*R*^2^ = 0.010, *p* = 0.141), supporting the claim of Epstein et al. (1996) that, to a certain extent, rational and experiential processes are independent.

Also no correlations were found between REI-10 scores and performance of the conjunction fallacy (*N* = 219, correlation coefficient ρ = 0.022 for NFC; *N* = 217, correlation coefficient ρ = 0.026 for FI), so scores were categorized by SD for further analysis. REI-10 scores were coded as *high* if they were more than 1 SD above the mean, *medium* if they were within 1 SD of the mean, or *low* if they were more than 1 SD below the mean. When the rational scale scores were categorized by SD, however, no significant relationships were found among the rational scale scores with the conjunction fallacy (*N* = 220, χ^2^ = 2.611, degrees of freedom = 4, *p* = 0.625, and correlation coefficient ρ = -0.037). Participants with high rational scores did not perform significantly better than those with medium or low scores, and participants with medium rational scores did also not perform significantly better than those with low scores in the Linda problem. Regression analysis showed that no gradient increase, e.g., the analytical-rational thinking style, was positively correlated with their performance (*N* = 219, *R*^2^ = 0.001, *F* = 0.11, and *p* = 0.741 for NFC) and that no gradient decrease, e.g., the experiential-intuitive cognitive style, was positively correlated with their performance (*N* = 217, *R*^2^ = 0.001, *F* = 0.143, and *p* = 0.706 for FI) in the conjunction fallacy. Furthermore, Epstein et al. (1996) classified four independent types of thinking styles for processing information: *rational* (high rational and low intuitive), *intuitive* (low rational and high intuitive), *complementary* (high rational and high intuitive), and *poor* (low rational and low intuitive). Results showed that the participants’ performance on the conjunction problem were not related to any of the four types of the thinking styles (*N* = 169 ^1^, χ^2^ = 2.508, degrees of freedom = 6, *p* = 0.868, and correlation coefficient ρ = 0.037). It is indefensible to predict that reliance on the analytical-rational cognitive processing will account for avoidance of the conjunction fallacy and that reliance on the intuitive-experiential cognitive processing will account for commitment of the conjunction fallacy.

These findings preliminarily contradicted the CEST’s prediction that participants who are characterized by “rational thinking” with high scores on the NFC and low scores on the FI are more inclined to use Bayes’ deduction than other participants who are labeled by “intuitive thinking” with low scores on the NFC and high scores on the FI or by “poor thinking” with low scores both on the NFC and FI. Of course, this speculation may be reversed based on a critical assumption: rational vs. experiential processing is not a static variable, but rather may change as an individual has more diverse life experiences that impel him or her to question the value of experiential processing.

## CONCLUSIONS

So far there are basically two perspectives regarding how people cognize the world. The first perspective has been underpinned by some experiments (e.g., Epstein et al., 1996; Stanovich and West, 1998; De Neys, 2006) that people rely on fundamentally two contrasting cognitive systems: the rational-analytical (or the so-called executive analytic) system and the experiential-intuitive (or the so-called automatic-heuristic) system. The two systems provide people dual-processing modes of reasoning in order to help them cope better with daily judgment and decision making. On the contrary, unlike the advocation by the dual-process theorists mentioned above, the second perspective declares that actually humans and animals make inference under limited logic deduction, cognitive capacities, knowledge, and time about the world. Therefore, viewpoints from ecological, bounded rationality indicate that people inference by a family of algorithms based on a simple psychological mechanism so-called the pragmatic heuristics (e.g., “Take The Best” algorithm by Gigerenzer and Goldstein, 1996).

The two camps of rational versus experiential cognitive styles and pragmatic heuristics are still debating whether the famous conjunction fallacy is the consequence of people’s automatically intuitive responses or the consequence of some particular cognitive algorithms that people implement (see, Lu, under review, for a discussion). Although cognitive styles can be considered as a personality trait with stabile specialities and can be measurable with designated self-report, it is still difficult to distinguish when computative analytic processing or instinct experiential processing is used in people’s reasoning. The class of pragmatic heuristics based on ecological and bounded rationality is possibly the actual result of an attempt to regulate the inner psychological balance between the emotional (intuitive) and the cognitive (rational) cognitive styles.

The role that individual differences (e.g., causal reasoning, cognitive ability, cognitive reflection, numeracy measures, and rational and intuitive thinking styles) play on some other judgment and decision tasks or biases has been also widely investigated so far (among others Shiloh et al., 2002; Peters et al., 2006; Liberali et al., 2012). For instance, Peters et al. (2006) found that highly numerate individuals are more affective and precise on judgment and decision tasks. They also found that highly numerate individuals are less likely to be susceptible to framing effects. It is worth mentioning that Liberali et al. (2012) related the analytical-rational and experiential-intuitive thinking styles of the CEST to numeracy measures and cognitive reflection rather than to the conjunction fallacy. Generally their results indicated that different aspects of numeracy (i.e., computational skills, metacognitive monitoring, and understanding the gist of relative magnitude) predict different biases and fallacies. Similarly, Shiloh et al. (2002) related the analytical-rational and experiential-intuitive thinking styles of the CEST to normative-statistical responses and framing effects rather than to the conjunction fallacy. Their studies found that normative-statistical responses are correlated positively with rational thinking style, and negatively with experiential-intuitive thinking style. On the other hand, specific combinations of thinking styles, high rational/high intuitive and low rational/low intuitive, are the ones most prone to framing effects.

The present paper attempted to address the void of correlations between cognitive styles and the conjunction fallacy based on the CEST model’s assumption of two rational and experiential systems (Epstein et al., 1996). The results found that low significant correlations between participants’ underlying cognitive styles and the conjunction fallacy contradicted the assumption that those participants with low rational and high experiential traits are easier to commit the conjunction fallacy than the other groups. Therefore, the present study indicates that people either with analytical-rational or experiential-intuitive (or so-called automatic-heuristic cognitive style) cognitive style does not influence the propensity for committing the conjunction fallacy.

It is noteworthy that for our limited sample age, context variation, and the employment of the REI-10, the present findings on the correlations between cognitive styles and the conjunction fallacy should be regarded as preliminary. Specially to note that though it has been established that the revised version of the REI scale (see, Pacini and Epstein, 1999; the scales for analytical and intuitive thinking both consist of 20 items) shows psychometric properties that increase the reliability of the instrument than the REI-10, considering for a limited experiment time, the current study still continued to use the more simplified REI-10 to measure the rational-experiential cognitive style.

In addition, ranking and probability estimation are two response modes that are commonly used in the conjunction fallacy as well as other studies. Hertwig and Chase (1998) confirmed that the probability estimation mode is assumed to trigger an integrate computing model (compensatory strategy) and the ranking mode to trigger a heuristic model (non-compensatory strategy). Therefore, the ranking mode may lead to more heuristic thinking than the probability estimation mode to induce the conjunction fallacy. It is noted that in their studies, a self-report questionnaire was used to measure which strategy their participants used to estimate probabilities or rank orders. However, as an explicit schema, the self-report technique is criticized to be susceptible to conscious explicit processing, i.e., participants may alter their more experiential-intuitive responses on a questionnaire. On the contrary, REI technique, as an implicit schema, is believed to be able to avoid conscious explicit problem. Further research might examine whether the experiential-intuitive cognitive style is inclining to lead to the conjunction fallacy and the rational-experiential cognitive style to no conjunction fallacy when asking participants to rank the Linda statements’ orders rather than asking participants to estimate probabilities in the current Experiment.

Other further research might examine the possibility that shifts and interacts in information processing, take into account more closely on the concrete information procession for the underlying mechanisms of the conjunction fallacy phenomena, and develop quantitatively specified cognitive process models of behavioral intentions to account for judgmental biases.

## Conflict of Interest Statement

The author declares that the research was conducted in the absence of any commercial or financial relationships that could be construed as a potential conflict of interest.

## Footnotes

1 Participants whose scores on the NFC or FI equated to 3 (intermediate) were excluded from the data analysis because they entailed no inclinations.
